# Dendritic Cell-Induced Th1 and Th17 Cell Differentiation for Cancer Therapy

**DOI:** 10.3390/vaccines1040527

**Published:** 2013-11-21

**Authors:** Julia Terhune, Erik Berk, Brian J. Czerniecki

**Affiliations:** 1 Department of Surgery and Harrison Department of Surgical Research, University of Pennsylvania, Philadelphia, PA 19104, USA; E-Mails: jterhune@smail.umaryland.edu (J.T.); erik.berk@uphs.upenn.edu (E.B.); 2 Rena Rowan Breast Center, University of Pennsylvania, Philadelphia, PA 19104, USA

**Keywords:** dendritic cell vaccine, cancer, immunotherapy

## Abstract

The success of cellular immunotherapies against cancer requires the generation of activated CD4^+^ and CD8^+^ T-cells. The type of T-cell response generated (e.g., Th1 or Th2) will determine the efficacy of the therapy, and it is generally assumed that a type-1 response is needed for optimal cancer treatment. IL-17 producing T-cells (Th17/Tc17) play an important role in autoimmune diseases, but their function in cancer is more controversial. While some studies have shown a pro-cancerous role for IL-17, other studies have shown an anti-tumor function. The induction of polarized T-cell responses can be regulated by dendritic cells (DCs). DCs are key regulators of the immune system with the ability to affect both innate and adaptive immune responses. These properties have led many researchers to study the use of *ex vivo* manipulated DCs for the treatment of various diseases, such as cancer and autoimmune diseases. While Th1/Tc1 cells are traditionally used for their potent anti-tumor responses, mounting evidence suggests Th17/Tc17 cells should be utilized by themselves or for the induction of optimal Th1 responses. It is therefore important to understand the factors involved in the induction of both type-1 and type-17 T-cell responses by DCs.

## 1. CD4^+^ T-Cell Differentiation

Dendritic cells (DCs) regulate the activation of naive T-cells by the uptake and processing of antigens and presenting them on their cell surface as peptides bound to major histocompatibility complex molecules (MHC), which is considered antigenic “signal 1”. Together with peptide:MHC complexes, DCs also express co-stimulatory molecules that provide signal 2 [1]. The combination of signal 1 and 2 regulate the specificity and magnitude of the T-cell response. DCs also provide a third signal (signal 3) to the T-cells, which is in the form of cytokines. Signal 3 determines the type of T-cell response that is elicited [2,3].

Different CD4^+^ T-helper cell populations are recognized based on their function and cytokine production [4,5,6,7]. Much of the early work in characterizing these populations and the conditions that favor their differentiation was performed on the T-helper (Th) 1 and Th2 subsets, as these were the first two populations described. Type-1 polarized CD4^+^ T-cells provide protection against intracellular infections, produce high levels of IFNγ and express the transcription factor, T-bet [8], while Th2 cells express the transcription factor, GATA-3 [9], produce IL-4, IL-5 and IL-13, enhance humoral immunity and protect against helminthes infections [10]. The production of interleukin (IL)-12p70 by DCs during the priming of CD4^+^ T-cells results in the induction of Th1 cells [11]. In contrast, the secretion of IL-4 during CD4^+^ T-cell priming results in the generation of Th2 CD4^+^ T-cells. 

More recently, regulatory CD4^+^ T-cells were described, which are recognized by the expression of the transcription factor, Foxp3, are dependent on IL-2 signaling and regulate immune responses by inhibiting T-cell proliferation and function [12]. Recently, a new population of CD4^+^ T-cells was described based on the expression of the transcription factor, retinoic acid-related orphan receptor (ROR)-γt and the production of IL-17A, IL-17F and IL-21 [7,13]. Th17 cells play a role in the immune response against extracellular bacterial and fungi infections and have been associated with various autoimmune diseases [14,15,16], as well as cancer [17].

The conditions that polarize a particular T-cell subset (e.g., IL-12 and IL-4) inhibit the differentiation of the other T-helper cell subsets, further enhancing the desired response. The presence of IL-12 during T-cell priming not only induces Th1 differentiation, it also inhibits Th2 differentiation, while the presence of IL-4 during priming regulates Th2 differentiation while preventing Th1 development. Furthermore, IFN-γ, produced in type-1 responses, downregulates IL-4 expression, thereby minimizing Th2 development [18], while IL-4, produced in type-2 responses, blocks expression of IL-12R, preventing Th1 development [19,20]. Similar to the effect of IL-12 on Th2 development and IL-4 on Th1 development, both IL-12 and IL-4 have been shown to prevent Th17 differentiation [21,22]. Likewise, the expression of transcription factors specific to a T-helper cell subset can also prevent the differentiation of other subsets. Th2 cells transduced to express T-bet developed into Th1 cells that produced IFNγ and lost their IL-4 and IL-5 production [8]. Conversely, the expression of GATA-3 by CD4^+^ T-cells prevented the production of IFNγ by T-cells primed under Th1-inducing conditions [23]. Furthermore, expression of T-bet prevented the generation of IL-17 secreting T-cells in a mouse model [24].

## 2. Requirements for Differentiation of Th17 Cells

Th1 cell differentiation has been well-described for years and is fairly straightforward: their differentiation is promoted by inflammatory environments that contain intracellular pathogens, such as viruses and some bacteria and protozoans and can also be induced by the presence of cytokines during T-cell priming; IL-2 and IL-12, in particular, synergize to promote IFNγ-secreting Th1 cells [25,26,27].

Th17 differentiation is not nearly so simple. Because IL-17 producing CD4^+^ T-cells are important in inflammatory and autoimmune diseases, many studies have explored the factors required for the differentiation of Th17 cells and the cellular origin of these cells; see Table 1 for a summary of the literature discussing the role of cytokines in human Th17 cell differentiation. 

The origin of human Th17 cells has received a fair amount of controversy, since initial studies were unable to show the differentiation of Th17 cells from circulating CD45RA^+^CD45RO^−^ (naive) CD4^+^ T-cells [28,29]. Cosmi and colleagues examined Th17 differentiation in detail and showed that IL-17-producing cells exclusively originated from CD161^+^ precursor cells [28]. Since naive circulating T-cells in adults are CD161^−^, this observation provides an explanation for the inability to detect Th17 development from naive CD4^+^ T-cells. In contrast, naive CD4^+^ T-cells derived from umbilical cord blood (UCB) or the thymus express CD161, and these naive CD4^+^CD161^+^ T-cells can be induced to differentiate into Th17 cells [28]. It was subsequently shown that all IL-17-producing T-cells, as well as precursors to IL-17 producing cells express CD161, which was, in part, regulated by the transcription factor, ROR variant 2 (RORC2), the human ortholog of mouse RORγt [30,31].

**Table 1:** Summary of the literature for cytokines involved in human Th17 cell differentiation.

Cytokines	Effect on human Th17 cell differentiation
IL-1β	*Positive effect on Th17 cell differentiation*
	Acosta-Rodriquez; Nature Immunology, 2007 [22]
	van Beelen; Immunity, 2007 [29]
	Manel; Nature Immunology, 2008 [32]
	Zielinski; Nature Letters, 2012 [33]
	Kryczek; Blood, 2009 [34]
	Wilson; Nature Immunology, 2007 [35]
	Volpe; Nature Immunology, 2008 [36]
IL-6	*Positive effect on Th17 cell differentiation*
	Acosta-Rodriquez; Nature Immunology, 2007 [22]
	Manel; Nature Immunology, 2008 [32]
	Zielinski; Nature Letters, 2012 [33]
	Volpe; Nature Immunology, 2008 [36]
	*Not necessary for Th17 cell differentiation*
	van Beelen; Immunity, 2007 [29]
	Kryczek; Blood, 2009 [34]
	Wilson; Nature Immunology, 2007 [35]
	Evans; Proc. Natl. Acad. Sci., 2007 [37]
IL-23	*Positive effect on Th17 cell differentiation*
	van Beelen; Immunity, 2007 [29]
	Manel; Nature Immunology, 2008 [32]
	Zielinski; Nature Letters 2012 [33]
	Kryczek; Blood, 2009 [34]
	Wilson; Nature Immunology, 2007 [35]
	Volpe; Nature Immunology, 2008 [36]
	*Necessary for effector function of Th17, but not for differentiation*
	Veldhoen; Immunity, 2006 [38]
	Elson; Gastroenterology, 2007 [39]
TGF-β	*Positive effect on Th17 cell differentiation*
	Manel; Nature Immunology, 2007 [32]
	Volpe; Nature Immunology, 2008 [36]
	*Negative effect on Th17 cell differentiation*
	Acosta-Rodriquez; Nature Immunology, 2007 [22]
	Wilson; Nature Immunology, 2007 [35]
	Evans; Proceeding of the National Acadamy of Science USA, 2007 [37]
	*Not necessary for Th17 cell differentiation*
	van Beelen; Immunity, 2007 [29]
	Kryczek; Blood, 2009 [34]

### 2.1. IL-23

Initial studies showed a correlation between IL-23 expression and the presence of Th17 cells, suggesting a prominent role for this cytokine in the differentiation of naive CD4^+^ T-cells into Th17 cells [40,41]. Subsequent studies have shown that IL-23 is required for Th17 cell effector function, expansion and survival, but not for their differentiation [34,39]. However, there are a number of studies that demonstrate that IL-23 is a critical component of the cytokine milieu supporting Th17 differentiation [29,32,33,34,35,36]. IL-23 by itself has been shown to be capable of supporting the differentiation of naive human CD4^+^ T-cells into Th17 cells [35]. Furthermore, DCs stimulated with a derivative of bacterial peptidoglycan (PGN) produce IL-23 and IL-1 and stimulate the development of Th17 cells, which could be inhibited by blocking IL-23 [29]. Although the precise role of IL-23 in Th17 cell differentiation remains to be fully defined, the data suggest that IL-23 has a valuable role in the survival and expansion of Th17 cells and might also play an important part in the differentiation of (human) Th17 cells.

### 2.2. Transforming Growth Factor-β

Mouse studies showed that the activation of CD4^+^ T-cells under inflammatory conditions in the presence of TGFβ and IL-6 results in the differentiation of Th17 cells [34,42]. In humans, however, the requirement for TGFβ in the differentiation of Th17 cells is still unclear. Various studies using Th17-associated cytokines revealed no role for TGFβ in the differentiation of human CD4^+^ T-cells into Th17 cells, and some found that TGFβ had a negative effect on Th17 differentiation. These studies showed that the differentiation of Th17 cells was dependent on the cytokines, IL-6, IL-1β and IL-23, alone or in combination [22,35]. However, recent data showed that TGFβ, in combination with IL-23, IL-1β and IL-6, was indispensable for driving the development of IL-17 producing human CD4^+^ T-cells. [32,36]. The observed contradictory roles for TGFβ in the development of human Th17 could be due to difference in the concentration of TGFβ used by the different research groups, with lower concentrations (1 ng/mL) being favorable for Th17 cell development and higher concentrations (5–10 ng/mL) being unfavorable [22,32,35,36,37,38,39,40,41,42].

### 2.3. Interleukin-6

IL-6, as mentioned above, is critical for mouse Th17 cell differentiation, but, like TGFβ, its role in human Th17 cell differentiation has been less clearly defined. Several of the studies discussed above showed that IL-6 is necessary for Th17 cell differentiation in concert with other cytokines [22,32,33,36]; however, there is considerable data demonstrating that it is not required [29,34,35,37]. Treatment of patients with rheumatoid arthritis (RA) with antibodies against the IL-6 receptor resulted in the skewing of T-cell differentiation towards Foxp3^+^ Tregs and away from Th17 cells [43]. Interestingly, IL-6 signaling induces the rapid upregulation of the IL-23 receptor on CD4^+^ T-cells, leading to Th17 cell induction, suggesting that IL-23 might play a role in the differentiation of Th17 cells [44]. 

### 2.4. IL-1β

IL-1β is frequently cited as one of the “pro-inflammatory” cytokines that either induces or enhances the differentiation of human Th17 cells [22,29,32,33,34,35,36]. This cytokine does not appear to be involved in the differentiation of Th17 cells in mice; however, the literature in humans supports the notion that it is a necessary component in human Th17 cell development.

### 2.5. Tc17 Cell Differentiation

While most studies focus on the development of Th17 cells, it appears that the cytokines required for the differentiation of IL-17-producing CD8^+^ T-cells (Tc17) are similar to those for Th17 differentiation. Several mouse studies have shown that the stimulation of splenocytes or isolated CD8^+^ T-cells in the presence of recombinant IL-6 or IL-21 strongly reduced IFNγ production by CD8^+^ T-cells. The addition of TGFβ further inhibited the Tc1 differentiation by blocking granzyme B expression and upregulating IL-17 production [45,46,47]. Furthermore, CD8^+^ T-cells activated in the presence of IL-6 and TGFβ expressed elevated levels of the transcription factor, RORγt [47]. 

In humans, patients with *Helicobacter pylori* infection show an increased expression of IL-23, which resulted in elevated production of IL-17 by CD8^+^ T-cells, as well as CD4^+^ T-cells, suggesting a role for this cytokine in Tc17 development [48]. In a human hepatocellular carcinoma study, it was shown that monocytes/macrophages recently activated in the tumor-microenvironment efficiently induced the development of Tc17 cells and that this development could be blocked by antibodies directed against IL-1β, IL-6 and IL-23 [49], suggesting that TGFβ is not required in humans for Tc17 development. Interestingly, a large percentage of these Tc17 cells also produced IFNγ.

## 3. Th1 and Th17 Cells in Autoimmunity and Cancer

### 3.1. Autoimmunity

Traditionally, autoimmune diseases have been associated with self-reactive hyperactive Th1 cells. However, mice lacking functional IL-12p70, lacking IFNγ or deficient in IFNγR signaling still developed certain autoimmune diseases. These paradoxical observations were resolved by the discovery of a new cytokine, IL-23, which is comprised of an IL-23p19 subunit and the IL-12p40 subunit, which it shares with IL-12p70 [50]. Experiments with mice lacking the IL-23p19 subunit, which are deficient in IL-23, but produce functional IL-12p70, revealed that these mice are resistant to the induction of experimental autoimmune encephalitis or collagen-induced arthritis, demonstrating the role of IL-23 in the pathogenesis of autoimmune diseases [51]. 

IL-23 is critically involved in maintaining the effector function of Th17 cells, and thus, the evidence linking IL-23 and autoimmune disease led to the association of Th17 cells and autoimmunity. This assertion has been supported by the detection of elevated IL-17 levels in the synovial fluid from rheumatoid arthritis (RA) patients [52], as well as in the serum of patients with inflammatory bowel disease [53]. Furthermore, models using IL-17-deficient mice or in which IL-17 was blocked by antibody treatment showed reduced inflammation and disease severity in rheumatoid arthritis and experimental autoimmune encephalomyelitis (EAE) models, further linking IL-23, IL-17 and autoimmune disease [14,16,54,55,56].

### 3.2. Cancer

T lymphocytes, both CD4^+^ and CD8^+^, are critical mediators in the immune system’s elimination of transformed cells. However, most studies have focused on differently polarized CD8^+^ T-cells, as these cells were considered the effectors, while CD4^+^ cells were thought to be supporting cells. CD8^+^ T-cells are infamous for their ability to lyse infected and transformed cells via the perforin/granzyme B pathway and the Fas/FasL pathway, earning them the reputation as the primary anticancer T-cells. CD4^+^ T-cells, on the other hand, were thought of only as support cells that would prime and sustain CD8^+^ T-cells and activate macrophages. However, it is now clear that CD4^+^ T-cell can kill tumor cells through direct cell contact via FasL- and TRAIL-dependent pathways, as well as through the perforin/granzyme B pathway, which is classically associated with cytotoxic CD8^+^ T-cells [57,58,59]. CD4^+^ T-cells also regulate the production of chemokines and, thereby, the attraction of cytotoxic CD8^+^ T-cells and other immune cells. Additionally, while it has been demonstrated that primary cytolytic CD8^+^ T-cell responses can be generated without CD4^+^ T-cells, CD4^+^ T-cells are necessary for the generation of CD8^+^ memory T-cell responses and the ability to rapidly and effectively extinguish future antigen challenges [60,61]. Taken altogether, T-helper cells have an integral role in the host defense against malignancy, and their incorporation into immunotherapy regimens is critical to the long-term success of such treatments.

Th1 cells are considered the primary T-helper cell subset involved in antitumor responses; they have been associated with anti-tumor responses in mouse models, achieved in part by their secretion of IFNγ. IFNγ has a myriad of functions in the immune system’s ability to control the growth of or eliminate tumors, notably the recruitment and activation of cells of the innate immune system and enhancing the production of anti-tumor chemokines [62,63]. IL-12p70, which strongly promotes the differentiation of type-1 T-cells, enhances IFNγ and granzyme B production, prolongs T-cell survival and enhances immune recognition of tumor antigen-expressing cells [64,65,66]. These factors together demonstrate why Th1 cells have been considered the premier cell to include in cancer immunotherapy regimens for most of the last decade. 

The role of Th17 and IL-17 producing cells in cancer, on the other hand, is controversial. Human cervical tumor cells transfected to express IL-17 were shown to have enhanced growth when transplanted into nude mice [67]. Furthermore, mice lacking IL-17 showed a reduced growth of B16 melanoma tumors and MB49 bladder carcinomas, suggesting a role for IL-17 in promoting tumor growth. Conversely, the growth of the tumors was enhanced in mice lacking IFNγ [68]. High levels of IL-17 producing cells in human breast cancer has been associated with decreased disease-free survival and tumor growth [69,70]. The elevated levels of Th17 cells in breast tumor tissues appears to be a result of increased levels of IL-23, due to tumor-produced prostaglandin E_2_ (PGE_2_) [71]. In colorectal tumor tissues, the production of IL-22, one of the cytokines produced by Th17 cells, was enhanced compared to normal tissue and correlated with enhanced IL-23 levels. IL-22-producing tumor-infiltrating lymphocytes (TILs) promote tumor growth and metastasis in a mouse model when co-transplanted with tumor cells [72]. Furthermore, while IL-12 has been shown to promote the infiltration of cytotoxic T-cells into tumors, IL-23 was shown to inhibit the migration of cytotoxic T-cells to tumor tissue and promote angiogenesis [73]. 

In contrast, some recent studies suggested an anti-tumor role for Th17 cells and IL-17 producing CD8^+^ T-cells. Muranski and colleagues showed that adoptive transfer of tumor-specific Th17 cells could be an efficient treatment of established tumors [74]. The Th17 cells showed a survival advantage over other transferred T-cells, and the anti-tumor immune response appeared to be dependent on IFNγ production [74]. The Dong group showed that adoptive transfer of tumor-specific Th17 cells resulted in robust activation of tumor-specific cytotoxic T-cells, and the anti-tumor response elicited was dependent on the Th17 cell-mediated production of CCL20 [75]. The increased chemokine secretion resulted in the enhanced attraction of DCs, T-cells and other leukocytes to the lungs and an increased activation of cytotoxic CD8^+^ T-cells. Interestingly, the transferred Th17 cells showed superior anti-tumor responses compared to transferred Th1 cells, and the Th17 cells retained their phenotype *in vivo* [75]. Another study evaluating anti-tumor responses in RORγt-deficient mice showed that reduced presence of Th17 cells in the tumor microenvironment resulted in enhanced tumor growth. This effect could be counteracted by adoptive transfer of Th17 cells [76].

A study in melanoma patients showed that vaccination with cell lysate-pulsed DCs resulted in an increase in both Th1 and Th17 cells in the peripheral blood. The increase in effector cells resulted in an enhanced immune response to tumor-antigen, as measured by delayed-type hypersensitivity (DTH) reaction and correlated with enhanced survival [77,78]. However, it is unclear whether the Th17 cells have a direct effect on tumor regression or an indirect effect by the attraction of type-1 cells. In ovarian cancer, the presence of Th17 cells in tumor tissue is positively correlated with effector cells and negatively associated with T_reg_ infiltration [38]. IL-17 synergized with IFNγ to enhance the production of the Th1-associated chemokines, CXCL9 and CXCL10, leading to an increase in effector cell infiltration. Together, these data suggest that Th17 cells might play an indirect role in tumor immunity through the attraction and activation of effector (Th1/Tc1) cells. See Table 2 for a summary of the literature discussed above regarding the pro-versus anti-tumor effect of Th17 cells.

**Table 2:** Highlights of the literature reviewing the pro- *versus* anti-tumor effect of Th17 cells.

**Pro-tumor**	Human cervical tumor cells overexpressing IL-17 have enhanced growth in nude mice [67]. Mice lacking IL-17 had reduced growth of melanoma and bladder tumors, and the growth of tumors was enhanced when IFNγ was lacking [68]. High levels of IL-17-producing cells associated with decreased disease-free survival [69] and increased tumor growth in breast cancer [70]. Evidence that tumors produce PGE2, which increases IL-23, which, in turn, enhances presence of Th17 cells in breast cancer [71]. In a colorectal cancer mouse model, transfer of IL-22-producing tumor-infiltrating lymphocytes with tumor cells promoted tumor growth and metastasis [72]. IL-23 is overexpressed in local tumor environment of human colon cancer patients and in mice has been shown to increase angiogenesis and inhibit migration of cytotoxic T-cells [73].
**Anti-tumor**	In ovarian cancer, the presence of Th17 cells is positively correlated with effector cells and negatively associated with Treg infiltration [38]. Adoptive transfer of tumor-specific Th17 cells can actually eradicate established melanoma tumors; Th17 cells showed survival advantage over other transferred cells, and the anti-tumor effect was dependent upon IFNγ production [74]. IL-17-deficient mice more susceptible to lung melanoma, and the adoptive transfer of tumor-specific Th17 cells prevented tumor development; transferred Th17 cells showed superior anti-tumor immunity as compared to transferred Th1 cells; the Th17 cell-mediated anti-tumor response was dependent on Th17 cell-produced CCL20 [75]. RORγt-deficient mice had reduced numbers of Th17 cells in the tumor microenvironment, and this led to enhanced tumor growth; adoptive transfer of Th17 cells reversed this effect [76].

Several groups have examined the adoptive transfer of IL-17 producing CD8^+^ T-cells. Similar to Th17 cells, the transferred Tc17 cells showed an enhanced survival compared to other transferred cells, which was associated with enhanced expression of IL-7Rα [45]. Furthermore, the anti-tumor effects of Tc17 cells appeared to be dependent on IFNγ produced in the tumor microenvironment, although it is not clear whether this IFNγ is produced by the Tc17 cells, after *in vivo* conversion into Tc1-like cells [45], or if it is produced by other cells in response to the Tc17 cells [79]. The mechanism by which Tc1 and Tc17 cells inhibit tumor growth appear to be different [79], and while Tc1 cells are more efficient than Tc17 cells, the latter might be important for the attraction of Th1/Tc1, as well as monocytes and neutrophils to the tumor [80]. On the other hand, while some studies showed that Tc17 cells lack granzyme B expression and cytolytic capacity, Tc17 cells might acquire IFNγ production (IL-17^+^/IFNγ^+^ CD8^+^ T-cells), granzyme B and FasL expression, as well as cytolytic ability after adoptive transfer or under the influence of IL-12 [81,82].

Although there is evidence suggesting a pro-tumor role for Th17 cells, accumulating data supports the notion that Th17 cells play an important anti-tumor role, particularly in the context of immune responses with combined Th1/Th17 properties and IFNγ production.

## 4. Dendritic Cells for Cancer Immunotherapy

Due to the ability of DCs to regulate immune responses, much effort has been put in the generation of DC-based therapies for the treatment of various diseases, such as cancer and autoimmune disease, as well as treatment for organ transplantation [83]. In 2010, the Food and Drug Administration approved the use of a cellular vaccine, Provenge (Sipuleucel-T), for the treatment of hormone refractory prostate cancer. This DC-containing vaccine improved overall survival in patients, but failed to induce tumor regression or to prolong time to disease progression, possibly due to the lack of mature DCs. These results highlight the feasibility of DC-containing cellular vaccines in the treatment of cancer, but also show the need to improve DC-maturation protocols [84,85]. The safety of DC-based therapies for the treatment of cancer has been demonstrated in clinical trials for the treatment of melanoma, lymphoma and renal cell carcinoma and breast cancer [86,87,88,89,90]. DC vaccines have also been proposed for diseases other than cancer, such as auto-immune diseases and tissue transplantation, in particular, based on their ability to induce peripheral tolerance via T_reg_ cells specific for auto-antigens [91,92,93,94]. 

Several broad requirements can be defined for the generation of suitable DCs for vaccination: first, the source of the DCs should be such that large numbers of clinical-grade DCs can be easily made. Secondly, the DCs should be matured in such a way that they express the desired co-stimulatory receptors and produce the appropriate cytokines and chemokines in order to effectively activate and polarize the T-cell response. Thirdly, the DCs should express the appropriate chemokine receptors and integrins to migrate to lymphoid organs or to specific tissues to interact with T-cells and induce the desired type of T-cell response. Each of these requirements, once a barrier to successful DC vaccines, has been extensively studied and systematically addressed over the past two decades, leading to huge gains in this field.

The first obstacle was overcome by the finding that monocytes, which are abundantly present in peripheral blood, can be induced to become immature dendritic cells (iDCs) upon culturing in the presence of granulocyte-macrophage colony-stimulating factor (GM-CSF) and IL-4 [95,96]. Early protocols devised for the generation of DCs from monocytes took up to seven days in culture [96,97], but a desire for more rapid maturation for clinical applications and a concern that DCs may become “exhausted” and unable to produce T-cell polarizing cytokines [98] led to the development of protocols with shorter maturation periods [66,99]. One such protocol developed by our lab for the generation of type-1 polarized DCs required only two days in culture and resulted in mature DC1s capable of T-cell sensitization [66]. Furthermore, this combination of GM-CSF and IL-4, followed by IFNγ, and, then, lipopolysaccharide (LPS) exposure also resulted in a second burst of IL-12 production upon interaction with CD40 ligand, suggesting that the key Th1 polarizing cytokine would be present at the time of DC-T-cell interactions [100]. The remaining requirements can be satisfied by using various combinations of inflammatory mediators during the time of dendritic cell maturation and will be discussed in detail.

Dendritic cells are exposed to countless signals in the body, leading to their maturation and influencing their polarization; replicating this *ex vivo* from monocytes requires precise application of inflammatory mediators, both innate and foreign. Maturing DCs with tumor necrosis factor-α (TNF-α) and cytokines, such as IL-1 and IL-6, leads to efficient upregulation of co-stimulatory molecules (CD83, CD86 and CD58), which are indicative of a mature DC phenotype [101]. Additionally, human peripheral blood monocytes treated with Toll-like receptor (TLR) agonist lipopolysaccharide (LPS) (a TLR4) and peptidoglycan (PGN) (a TLR2 ligand) express markers of DC maturation, CD40, CD83, CD86 and HLA-DR [102], demonstrating that both innate inflammatory markers and inflammatory signals from bacteria can lead to DC maturation.

There are numerous *in vitro* protocols to generate different DC subsets that induce specific T-cell responses, many of which rely on the usage of inflammatory mediators, such as TNFα and type-1 interferons in combination with ligands for pattern recognition receptors, such as the TLR-4 ligand LPS or the TLR-3 ligand poly-I:C [101]. For instance, the induction of a type-1 T-cell response is frequently sought for its antitumor properties, and employing dendritic cells polarized into type-1 DCs (DC1s) to achieve this is well described. A key characteristic of DC1s that is necessary for the induction of type-1 T-cell responses is the production of IL-12p70 [103,104]. This DC1 phenotype can be achieved by cross-linking of CD40 with its ligand, CD154 (CD40L) [105,106,107,108,109,110], or by exposing immature DCs (iDCs) to various inflammatory molecules during their maturation [105,111,112]. High levels of IL-12p70 production by mature DC1s requires at least two signals [113] and is enhanced by IFNγ [114]. In contrast, induction of the related cytokine, IL-23, by DCs in response to microbial stimuli is not enhanced by IFNγ [114] and can be elicited with exposure to only one inflammatory mediator [115]. CD40-CD40L interactions, though, do stimulate the secretion of IL-23 [50,112,116]. 

The ability to traffic to inflamed tissues to take-up antigen, promptly exit and traffic to secondary lymph organs to prime T-cells is a key characteristic of antigen-presenting cells. For immunotherapy applications, however, DCs would ideally traffic directly to a lymph node to interact with T-cells upon transfer into the patient. Maturation of DCs with inflammatory agents, such as LPS or TNF-α plus CD40 ligand, results in rapid down regulation of CCR1, CCR5 and CXCR1, chemokine receptors important for trafficking to inflamed tissues [117]; minimal expression of these chemokine receptors is desired so that the transferred DCs do not migrate to inflamed tissues. Use of the same maturation agents leads to efficient upregulation of the chemokine receptors, CCR7, CXCR4 and CCR4; two ligands for CCR7, namely CCL19 and CCL21, are known to be expressed throughout the lymphatic system [117,118]. Furthermore, CCR7 and CXCR4 are known to also be expressed on naive T-cells, further supporting the co-localization of mature DCs and naive T-cells to allow for their interaction [117]. Additionally, CCR7 signaling is known to support DC survival, dendrite process formation and antigen uptake, leading to more efficient T-cell responses [119]. Together, these results demonstrate how maturation of DCs with inflammatory cytokines and/or bacterial products promotes chemokine receptor expression that allow appropriate transit towards secondary lymphoid organs.

### 4.1. Toll-Like Receptor (TLR) Agonists as Maturation Agents for DC1 and DC17s and Polarization of T-Cell Responses

Toll-like receptor agonists, as mentioned previously, are a class of inflammatory mediators that can be used to mature DCs and polarize their cytokine production, ultimately impacting the type of T-cell response elicited. Early work in this field identified the TLR4 ligand, LPS, as a potent inducer of IL-12p70 and IP-10/CXCL10 by dendritic cells, attracting monocytes and Th1 cells and further enhancing the type-1 immune responses [102]. TLR2 agonists, on the other hand, induced IL-23p19 production, which is now known to be associated with type-17 immune responses [102]. Recent work on a subset of DCs, called inflammatory DCs (infDC), demonstrated that the TLR2/TLR1 ligand, Pam3Csk4, led to a DC cytokine profile capable of inducing Th17 cell differentiation from naïve CD4^+^ T-cells [120]. Interestingly, while lipopolysaccharide (LPS) derived from *E. coli* typically signals through TLR4 and induces a predominately Th1 response, it has recently been shown that an atypical LPS from *P. gingivalis* signals through TLR2 and induces slightly more IL-23 production than the typical LPS, demonstrating remarkable specificity [121]. 

The bacterial cell-wall component, peptidoglycan (PGN), is a TLR2 ligand and stimulates DCs to secrete IL-23 and IL-1β, resulting in Th17 cell induction [29]. The DC17 polarizing effect of PGN was regulated through metabolism into the nucleotide oligomerization domain 2 (NOD2)-ligand muramyl dipeptide (MDP). Stimulation of iDCs with TLR ligands in the presence of MDP resulted in the production of IL-23 and IL-1, promoting IL-17 producing T-cells [29]. Finally, culturing human CD4^+^ T-cells with anti-CD3 antibodies plus monocytes with the TLR ligand, LPS, has been shown to lead to the induction of IFNγ-secreting cells, IL-17-secreting cells and a third population of dual-secreting cells [42], which could be the most desirable outcome for cancer applications. 

### 4.2. Use of Prostaglandin E_2_ as a DC Maturation Agent

PGE_2_ became a component in many DC maturation protocols after it was demonstrated that the expression of CCR7 on *in vitro* matured monocyte-derived DCs was enhanced by maturation in the presence of PGE_2_. However, it has since been shown that DC expression of CCR7 can be induced in the absence of PGE_2_ [122]. The advantage of maturation protocols without PGE_2_ is that the DCs appear to produce higher levels of the type-1 polarizing factor, IL-12p70 [123,124]. Furthermore, PGE_2_-matured DCs have been shown to induce Th2 cells [125,126], and PGE_2_ regulates the production of the immune inhibitory cytokine, IL-10 [125]. Also of importance, PGE_2_ has been associated with enhanced T_reg_ differentiation, function and attraction via secretion of CCL22 [127,128,129], though some studies also suggest a role for PGE_2_ in the induction of Th17 cells [71,130,131].

### 4.3. Interleukin-4

Most protocols for the generation of DCs from peripheral blood monocytes rely on the use of GM-CSF and IL-4. However, some recent studies promote the use of IL-15 instead of IL-4 for the generation of T-cell activating DCs [132,133]. While these “IL-15-DCs” resemble Langerhans cells [134], activation of these DCs with TLR agonists results in the generation of both Th17 and Th1 cells [135].

**Figure 1:**
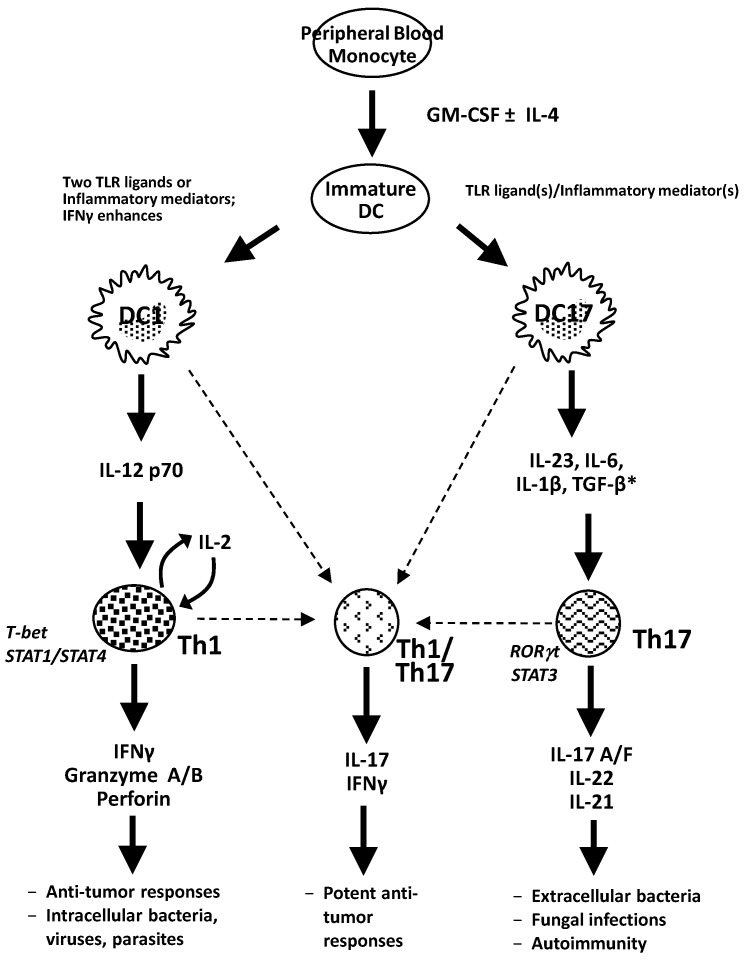
Dendritic cell induced Th1 and Th17 immune responses. Toll-like receptor (TLR) ligands are used to mature dendritic cells, and depending on the ligand(s) selected, type-1 dendritic cells (DC1s) or type-17 dendritic cells (DC17s) result. DC1s are characterized by the production of IL-12p70 and induce Th1 immune responses with interferon-γ, granzyme A/B, or perforin secretion. DC17 cells are characterized by the production of a number of cytokines, including IL-23, IL-6, IL-1β and TGF-β, and polarize Th17 immune responses with IL-17A/F, IL-22 and IL-21 production. The cytokines denoted with an asterisk (*) have been reported in the literature to have a role in inducing human Th17 cell differentiation, though there are conflicting reports, and it remains unclear precisely which cytokines are in fact necessary. Finally, there is a third population of T-cells that can be induced by dendritic cells that secrete both IFNγ and IL-17, though the exact mechanism of their differentiation and whether they are directly induced by DCs or are the result of a conversion from Th1 or Th17 cells has yet to be elucidated.

These data show that different maturation stimuli can be employed to regulate the level of IL-12p70 production by DCs and induce distinct types of T-cell responses, offering an opportunity to modulate the host immune response. Refer to Figure 1 for an overview of dendritic cell-induced Th1 and Th17 immune responses.

## 5. Conclusions

Dendritic cell-based immunotherapies hold much promise in manipulating the *in vivo* immune response to attack and eliminate malignancies. Ultimately, multiple subsets of DC may be needed in successful cancer vaccines. The selection of an appropriate maturation protocol for the DCs is of paramount importance: without the proper *ex vivo* culture conditions, the DCs may not be mature or capable of activating a lasting T-cell response. Furthermore, the specific agents selected for DC maturation will determine the type of T-cell response elicited and, therefore, must be carefully selected. We feel that producing DCs that can generate both Th1 and Th17 cells, in addition to CD8^+^ T-cells, will effectively utilize the anti-tumor properties of each of these cells and ultimately may lead to success in the treatment of any number of cancers.
